# Participatory Animation for Health Promotion in Digital-Based Health Interventions: Viewpoint on Methodology and Application

**DOI:** 10.2196/72737

**Published:** 2025-12-05

**Authors:** Essence Lynn Wilson

**Affiliations:** 1 Department of Psychology, California State University, Northridge, 18111 Nordhoff Street, Northridge, CA, 91330, United States, 1 (310) 946-2531

**Keywords:** digital-based health interventions, participatory methods, health determinants, animation, community-engaged research

## Abstract

Digital-based health interventions (DHIs), defined as health services delivered electronically, have demonstrated effectiveness in promoting health outcomes. However, DHIs often suffer from low user retention, a challenge attributed to limited attention to sociocultural determinants and insufficient user engagement strategies. This paper explores participatory animation (PA), a collaborative methodology that engages community partners in co-creating animated content, as a strategy to improve DHI retention and effectiveness. Drawing from existing literature, this viewpoint examines the theoretical foundations and practical affordances of PA for enhancing DHIs. We describe PA as a multistep production process that integrates participant-driven oral and visual design contributions into multimedia outputs for use in DHIs. Here, PA shows promise in producing engaging and culturally resonant content, with the potential to improve intervention uptake and sustain user engagement. Despite these affordances, PA remains underused in health research. Given the growing urgency to develop effective, equitable DHIs, PA offers a novel, community-informed approach for enhancing both design and implementation. This paper positions PA as a methodological frontier for DHI science.

## Introduction

Health scholars increasingly use digital-based health interventions (DHIs) to improve patient health outcomes through interactive, scalable formats [1]. These interventions encompass digitized health services such as virtual environments and personalized video content, designed to engage diverse populations and address complex health issues [2-4]. Indeed, DHIs have demonstrated effectiveness in increasing treatment uptake, such as for mental health and substance use cessation services [5,6].

Despite their widespread adoption, DHIs’ efforts in sustaining participant use have historically underperformed [7-9]. While many interventions demonstrate efficacy in improving health outcomes, often grounded in behavioral science frameworks [6,10], long-term participant retention remains limited [7-9]. Systematic reviews and meta-analyses across eHealth, mobile health, and broader DHIs consistently report moderate to small effect sizes, an outcome posited to implementation shortcomings and low user engagement efforts and user engagement [11-13].

The limited impact of many DHIs highlights gaps in their development, particularly in their ability to address the complex, multilevel determinants of health, often rooted in the interplay between physical, cultural, economic, and political environments [14,15]. However, many DHIs inadequately engaged with these broader influences, limiting their relevance to the target population they intend to serve target population [16]. As such, DHIs are experiencing low participant engagement, as reflected in consistently high attrition and low adherence rates [17-21].

Integrating participatory frameworks in DHI development offers a strategic means to address complex health determinants and improve intervention effectiveness. By fostering equitable collaboration between researchers and the communities they intend to serve, these practices democratize the research process and shift focus beyond individual behaviors to address broader determinants of health, a key consideration in DHI development [22]. Participatory approaches have demonstrated success in enhancing user engagement and retention, particularly within underserved populations [23-25]. They can also empower resource-limited communities by reducing barriers to achieving a healthier environment, making them especially valuable for addressing health disparities in historically marginalized groups [26].

*Participatory animation* (PA), situated within broader participatory research traditions (eg, written, observational, and visual), offers a distinct method for exploring complex health determinants and enhancing engagement with DHIs [12]. Drawing from the foundational work of Lomax and Smith [27], PA functions as both a methodology and cultural practice that seeks to illuminate the inherent and relational dialogical processes involved in the co-production of animation. Animation is defined as “any application that generates a series of frames, with each frame appearing as a modification of the previous one,” with each sequence or narrative determined by the designer(s) [28,29]. Evidence suggests that animation, when integrated into DHIs, can improve intervention outcomes, including increased patient knowledge [30] and more positive health attitudes and behaviors [31]. Moreover, animated outputs can be iteratively refined into evidence-based interventions, offering a degree of narrative flexibility and substantive participant representation that may not be achievable through other visual or digital modalities.

Drawing from available scholarship, this viewpoint considers PA a promising health promotion method. More specifically, this paper offers a perspective on PA as an emerging methodology for enhancing DHI outcomes, synthesizing theoretical foundations, reflecting on its practical applications, and proposing directions for future research. This paper substantiates DHI science in offering a unique and dualistic strategy for assessing complex health determinants and enhancing intervention features necessary for heightened patient prohealth activity. We discuss this method’s features, including context on when to use it, highlight core theoretical frameworks that support its ability to enhance DHI structures, and discuss logistical challenges to ensure transparency in research application. An innovative methodological intervention in health research, we ask the following research questions:

1. What is PA, and what are its unique affordances, including its theoretical foundations, for promoting prohealth outcomes within a digital context?
2. In addition to examining its affordances for health promotion, what are the potential challenges of using PA in health research?

## Literature Review

### Overview

Healthy People 2030’s aim, in line with society’s growing reliance on digital resources, is to “make electronic health information easy to understand and use [as this] is key to improving health and well-being” [32]. This proposal has two aims: (1) to encourage robust production of digital resources (eg, DHIs) to improve health and well-being, and (2) to use digital-based mediums that match the target population’s existing behaviors and attitudes to its technological capabilities, focusing on the affordances of animation for this paper. Here, we address these claims in the review that follows.

### Digital-Based Health Interventions

Emerging technologies offer substantial potential for expanding access to evidence-based health care, particularly through DHIs. These interventions, which are health services delivered through both formal and informal digital platforms, have been increasingly adopted across a variety of health domains. Research consistently shows that DHIs often outperform standard treatments in improving health outcomes [2]. Meta-analytic evidence supports their effectiveness in several areas, including mental health (Hedge G=0.40) [6], physical exercise promotion (mean difference=49) [33], and substance use and smoking cessation (log RR=0.77) [5].

Recent research highlights the potential of DHIs to influence both health behaviors and outcomes. One key advantage is their capacity to reach a broad and often hard-to-reach populations, including those with limited access to traditional health care services [34]. Another advantage is the low-cost per user model, which allows health care professionals and advocates to extend their reach and increase public access to care. Additionally, the private and user-directed nature of DHIs can reduce stigma-related barriers by allowing individuals to engage with health content discreetly, outside public or clinical settings [10].

While DHIs show promise in reducing health disparities by expanding their access to timely, technology-enabled care, their effectiveness is often undermined by poor user retention. High attrition rates remain a consistent challenge across DHI modalities [11-13,20]. For example, Kampmeijer et al’s systematic review of eHealth and mobile health interventions [13] among older adults reported that 25% of studies identified motivation-related barriers, including difficulty using new technologies. These retention challenges are frequently attributed to a limited understanding of users’ needs, including their technological comfort, motivational drivers, and contextual barriers [35]

### Assessing Complex Health Determinants

Enhancing user engagement in DHIs requires attention to factors beyond individual behaviors. The Bronfenbrenner socioecological model (SEM) offers a useful framework for examining multilevel influences that shape health outcomes, particularly among communities facing intersecting structural challenges. The SEM includes 4 primary levels: the individual level, which contains physical, cognitive, and emotional health factors; the interpersonal level, involving social networks and relationships [36,37]; the community level, which considers access to and navigation of institutions such as health care, higher education, and media; and the institutional or structural level, which addresses systemic forces such as economic conditions, political environments, and cultural norms [38]. By incorporating these interconnected layers, the SEM enables a more holistic approach to designing DHIs that resonate with users’ lived experiences and contexts.

Empirical applications of SEM demonstrate its use in shaping effective health strategies. For example, Byrd and McKinney [39] applied this model to guide clinical outreach and research initiatives that supported more inclusive campus climates and policies for university students. Building on this, Wilson et al [40] conducted a cross-sectional analysis to address mental health disparities among African American college students. Their findings highlighted the need for institutional policies that recognize race-based discrimination as a significant stressor and emphasized the importance of broader support systems, such as cultural affinity groups and strength-based evaluations of campus health services.

Applying a multilevel framework also informs the design of digital intervention strategies. Fish et al [41] developed Q Chat Space, a digital support platform for LGBTQ+ youth by accounting for contextual factors such as geographic location and family structures to optimize engagement. Similarly, Macheroni et al [42] assessed a survey of 10,000 European children, finding that perceived individual and social discrimination was negatively associated with self-efficacy in digital skill development. They recommend targeted intervention to address offline discrimination to promote uptake and effective use of digital tools. As we discuss in the “Exploring Participatory Frameworks for Health Promotion” section, participatory approaches, particularly PA, are well-positioned to support this goal by integrating community input and contextually relevant information into intervention design.

### User Engagement in DHI Structures

Perski et al [43] define user engagement as the extent of digital use, encompassing metrics such as volume, frequency, duration, and depth of digital interaction. Beyond these behavioral measures, engagement also includes subjective experience, such as attention, interest, and emotional investment, that contribute to sustained intervention use [44,45]. Despite the centrality of engagement to intervention success, the factors that enhance user engagement remain underexplored in DHI research. This gap is reflected in persistently high attrition rates and low adherence across [17-19,21].

Visual salience and contextually relevant design play a critical role in enhancing user engagement with DHIs [23]. Studies consistently show that the perceived attractiveness of an intervention positively influences user retention [46,47]. Among the most effective strategies are the integration of context-congruent animation and digital narratives, both of which have been shown to improve message uptake and promote prohealth behaviors [48-51].

Despite growing interest in DHIs, few interventions are specifically designed to reflect racial or cultural experiences, which can limit engagement among diverse populations [16]. Jones et al [52] identified several engagement-enhancing affordances when DHIs directly addressed racial stressors and incorporated culturally affirming elements, such as messages promoting racial pride. Embedding positive cultural context, such as racial identity and socialization or “positive verbal and non-verbal messages, actions, and beliefs, conveyed about [cultural factor, or in this case race] increasing understanding of the …ecology” can deepen ecological understanding and improve relevance to the target population.

### Exploring Participatory Frameworks for Health Promotion

Incorporating a participatory framework into DHI development can help address persistent challenges in user retention (Table S1 in Multimedia Appendix 1). Here, participatory practices can (1) assess formative data and enhance data triangulation to understand the scope of determinants, (2) design intervention components to increase engagement, and (3) expand networks for outreach by ascertaining ecological barriers and facilitators [53]. Specific to digitized spaces, community partners can collaborate among researchers and stakeholders for content design and provide iterative feedback on feasibility and acceptability [54,55]. For example, June and Aguilar’s case study of the Central Texas African American Family Support Conference [53] demonstrated how shared understanding and collective responsibilities between community partners and stakeholders fostered long-term engagement and program sustainability. Such frameworks are particularly effective in underserved populations, where socioecological complexity requires grounded, community-informed strategies. Indeed, community involvement, if not immersion, can lead to successful engagement and heighten prohealth efficacy outcomes [23,25].

Despite the potential of participatory approaches in technology-enabled interventions, few DHIs have fully integrated such methods [23]. A notable exception is MindLight*,* an anxiety prevention video game developed by Schoneveld et al [56] for school-age children. In this project, researchers collaborated directly with children diagnosed with anxiety disorders, co-creating monster character designs that were later incorporated into the game. The participatory design process not only acknowledged the children’s lived experiences, but also enhanced the game’s ability to capture and retain users. This intervention led to a statistically significant reduction in anxiety symptoms between pre-post test conditions, illustrating the value of embedding participatory elements in DHI development.

In this paper, we define a participatory framework as a methodological approach that centers equitable collaboration between researchers and community stakeholders, emphasizing co-design, iterative feedback, and shared ownership of knowledge production [25,26]. This definition draws from foundational community-based participatory research literature [14,26,57] and human-centered design strategies applied to digital health [54].

### Participatory Animation

#### Overview

This paper gives a perspective on PA as a unique opportunity to break down barriers in traditional health research. It highlights its potential as an innovative approach in health promotion geared towards comprehensive intervention design and implementation. Its future directions include refining intervention strategies based on community feedback to sustain engagement and enhance health outcomes.

More specifically, this work is best understood as a conceptually grounded synthesis, integrating existing literature with illustrative examples drawn from prior studies. While no new primary data were collected, the analysis was informed by participatory design principles and grounded in empirical case applications from the field.

#### Affordances

PA is a methodological and cultural practice centered on the co-creation of animated content [27]. This approach has two key affordances: (1) the co-production process illuminates inherent relational and dialogical processes between participants and researchers, and (2) the resulting animations can serve as multimedia tools to enhance DHI outcomes [48,58]. Indeed, animation as a participatory practice has been effective for digital health promotion, including areas in community wellness [59], building healthy interpersonal connections [60,61], developing culturally responsive therapeutic practices [62], and fostering safety and belonging for refugee children [63]

Animation as a medium illustrates a unique potential for community engagement. It is defined as “any application that generates a series of frames, with each frame appearing as a modification of the previous one,” where each sequence or narrative is determined by the designers [28,29]. Animation can break down the barriers of written text, making content accessible, and can be culturally tailored to reflect the lived experiences of specific target populations. Its iterative nature also allows for ongoing input and refinement, enabling participants to co-construct meaning through a combination of oral and visual storytelling.

The development of animated content can foster civic engagement by empowering community partners to share their experiences, perspectives, and ideas. PA brings together diverse forms of experiences, knowledge, and skills, creating opportunities to explore multilevel health determinants through collaborative design [26,57,64,65]. The production process integrates various artistic and linguistic forms, including creative writing through script development, sound and music composition, and visual design such as character modeling and background art. The multidimensional input not only amplifies participant voices but also enables researchers to gather intersecting forms of data, thereby enhancing the richness of analysis and opportunities for data triangulation.

PA can semiotically manifest an individual’s experience by empowering community partners to shape their self-presentation, offering narrative control [27]. Animation production can democratize the research process, countering traditional models in which participant voices are often filtered through external interpretation [66,67]. By fostering collaboration and mutual respect, PA can also serve as a mechanism for building trust between community partners and researchers [68]. For example, Lomax and Smith [27] used animation to capture children’s emotional experience during the COVID-19 lockdown. The use of participant-generated voiceovers preserved nuance, such as humor and tone, not easily conveyed through transcripts. Similarly, Massijeh [63] used animation as a tool for play and exploration with South Sudanese refugee children, enabling them to articulate ideas that may have been inaccessible through traditional methods. These examples highlight how PA can enrich data collection and enhance the relevance of formative assessment [69].

Indeed, participatory practices can help address the limitations of traditional qualitative methods like focus groups and interviews. Animation can enhance communication by capturing emotional nuance, symbolic meaning, and complex messages that may be difficult to articulate through verbal methods alone. It can also overcome language barriers, offering a meta-narrative format that converts ideas visually and universally. Vaughn and Jacquez [15] demonstrated this effectively in their community-based project with youth partners aimed at reducing violence and promoting neighborhood well-being. Their iterative process, involving story vignettes, storyboarding, voiceover recordings, and animated revisions, revealed facilitators that may have been overlooked through conventional research, such as the partners’ preference for visually appealing, short-form messaging tailored to peer audiences.

PA can be used to explain causal relationships and pathways, whether through multilingual assessments or empirical testing of its messaging effects. Offering multiple textual domains—visual, auditory, and narrative—this approach enables the exploration of complex social structures that may be inaccessible through more traditional or unidimensional methods. For instance, Janssen et al [70] documented how employees used animation to simulate organizational processes and support the adoption of shared services. Their participatory process created a direct pipeline from data collection to content creation, culminating in a film presented to stakeholders for evaluation. As a determinant of efficiency in the business setting, animation, in this case, is used as a participatory praxis to assess causal links while providing a product to engage clients.

Finally, PA also serves as an effective and timely tool for disseminating health messages to diverse community segments. Research indicates that animation can enhance DHI structures and enhance messaging, particularly through widely used digital devices, including smartphones, tablets, and social networking sites [30,31]. Vaughn and Jacquez [15] collaborated with community members to develop violence-prevention animations, which were strategically shared through social networking channels to maximize relevance and reach. This approach illustrates how PA can extend the lifecycle of community-driven interventions beyond the research setting and into everyday digital environments. Here, they co-produced violence-prevention animated content with residents and disseminated it to targeted social networking outlets.

Overall, PA can be understood along 2 key dimensions: as a process and as a product. As a process, this method involves iterative collaboration with community members through processes such as scripting, voiceover development, storyboarding, and visual design, a creative cycle that fosters trust, reflection, and shared meaning-making. As a product, the resulting animated content serves as a multimodal tool for intervention delivery, communication, or knowledge dissemination. This distinction is critical: the process facilitates engagement and data generation, while the product enhances message clarity and reach. Recognizing both dimensions helps researchers design PA projects that are both methodologically rigorous and practically impactful [27,48,59].

It is important to note that PA shares similarities with other participatory visual methods such as photovoice, participatory video, and theater. Scholars and practitioners have used these methods to center community voices, facilitate collective reflection, and aim to shift power, if not democratize, research processes. However, PA offers distinct affordances. Unlike photovoice, which relies on still imagery, or participatory video, which typically requires synchronous participation and access to equipment, animation allows for asynchronous collaboration. Here, community members can contribute ideas, voiceovers, or drawings through diverse and flexible timelines. Compared to theater, which foregrounds live embodiment and performance, PA can preserve anonymity and privacy, which may be especially valuable in stigmatized health contexts. Moreover, its multimodal nature—blending sound, image, text, and movement—makes it uniquely suited for communicating complex, layered health determinants across digital platforms. Indeed, these affordances specifically position PA as a scalable and context-sensitive approach for DHI development, particularly for and by communities with varying levels of technological and physical access.

#### Theoretical Foundation

PA is supported by multiple theoretical frameworks that underscore its value for advancing DHI research and practice (Table S2 in Multimedia Appendix 1). From a dialogical framework perspective, animation production unfolds through a series of phases that enable diverse modes of reflection and communication. Indeed, “much of the work [in animation development] occurs under closed doors and is made of incredibly complex intersubjective exchanges, negotiations, and contestations,” offering rich opportunities to collect and assess complex health determinants [71]. Within this framework, PA can help address what Bourdieu et al [72] termed the ‘paradox of communication’ or the difficulty of conveying lived experience within dominant linguistic or symbolic structures. As Frank argues [73], dialogical narratives are especially valuable for disrupting hegemonic frames and making space for alternative or marginalized forms of expression.

The multimodal nature of animations allows for the transmission of rich, layered information shaped by its creators. Frank’s dialogical narrative analysis [73] provides a useful lens for understanding this potential, emphasizing the value of examining tensions across different communication modes. His framework identifies 3 distinct publics that help explore the association between visual storytelling and community output evaluation. The first public comprises the core group of participants and researchers or filmmakers, fostering idiosyncratic relationships. The second public includes individuals outside the research project who clearly understand its context and have indirect connections, such as film viewers. The third public, a broader audience, views the film independently and lacks the opportunity for direct engagement or participation in an intersubjective process. This 3-tiered model supports a comprehensive exploration of animation’s communicative reach and highlights its potential to foster engagement and meaning-making across multiple levels of audience participation.

Animation alone is supported by numerous cognitive theories that can bolster DHIs’ structures. It is supported by the cognitive theory of multimedia learning proposed by Mayer and Moreno [74]. This theory posits that learners engage in three critical cognitive processes when exposed to multimedia content that incites auditory and visual sensations: (1) incoming verbal information to create text schema, (2) incoming visual information to form an image base, and (3) a combination of verbally and visually based models which enhance knowledge acquisition, a variable shown to encourage prohealth behaviors [75]. Cognitive scholars Berney and Bétrancourt [76] found this framework helpful for understanding animation’s empirical effects in enhancing participant learning across 50 studies compared to static illustration. These animations simultaneously presented verbal and visual materials that enhanced participant knowledge on the subject.

The social cognitive theory, developed by Bandura [75], further reinforces the value of animation in enhancing DHIs. This theory posits that individuals acquire knowledge not only through direct experience but also by observing others, whether through social interactions or mediated representations. When paired with the cognitive theory of multimedia learning, animations become a powerful tool for modeling behavior, allowing users to visualize actions, consequences, and strategies in ways that support observational learning. O’Day [77] elucidates animation’s effects in enhancing modeling structures, where teaching procedural information through dynamic events was more effective than static sequential images.

The cognitive load theory also supports the strategic use of animation in DHIs [78]. This theory emphasizes the limitations of working memory and the importance of minimizing extraneous cognitive demands to facilitate learning and long-term retention. Animation can reduce cognitive load by integrating visual and verbal information in a way that supports comprehension. For instance, animation that includes complementary text content congruent to the targeted message and signaling cues (eg, highlighting, arrows, and flashing symbols) helps direct user attention and reinforce key concepts [79,80]. These features enable users to process complex information more effectively, thereby improving message clarity and enhancing prohealth outcomes [81].

Overall, PA leveraged across numerous research capacities, contingent upon the researcher’s epistemological perspective. Researchers may opt for a critical, interpretative, practical, or positivist approach.

## Discussion

This paper explored PA as a novel methodological approach for improving DHIs, particularly in addressing persistent issues of low user engagement and retention. Through a synthesis of theoretical frameworks and empirical examples, we identified this method’s key affordances, including its potential to incorporate sociocultural determinants, foster participant co-creation, democratize research settings, and enhance message clarity through multimodal storytelling. While underused in the current DHI design, PA offers unique benefits for tailoring interventions to diverse and hard-to-reach populations and increasing their resonance and sustainability.

Effective implementation of PA requires careful consideration of several logistical challenges, particularly around data interpretation. Given the multimodal nature of the medium, there is currently no standardized approach for analyzing its components such as storyboards, voiceovers, or scripts, which can complicate methodological rigor. Existing scholarship has primarily explored PA within the context of health messaging, yet analytic strategies remain highly dependent on the researcher’s disciplinary orientation. For example, some may focus on disseminating co-created content through digital platforms to enhance message delivery, while others may treat the animation process as a data source, applying qualitative methods such as thematic or discourse analysis to examine its narrative and symbolic dimensions. This variability underscores the need for clearer guidance and methodological transparency in future applications [82].

Editing completed animated content for intervention refinement presents several challenges. Most iterative testing tends to occur during the storyboard phases, where collaborators co-develop a visual plan outlining the animation frame-by-frame. However, once production begins, making substantive changes can be labor-intensive and costly. Creating high-quality content, comparable to that produced by commercial studios, can cost up to $7000 per minute [83]. Costs vary depending on how labor is allocated; for example, budgets can be reduced by limited preproduction work, such as concept art, or by using prebuilt assets and lower-frame-rate animation tools. There are also cost-efficient alternatives to producing animation, such as Vyond, PowToon, or Canvas animation, all of which enable users to generate animated presentations with easy-to-use features. Meanwhile, university-community partnerships with student animators, apprenticeship (eg, Legends Animated), or with freelance animators can also offset costs [84].

Nonanimated media may be more appropriate for specific community segments. It is critical to acknowledge regional perspectives on animation when implementing PA. Community partners from European and North American regions may robustly perceive animation as an entertainment source for children [85]. This is reflected in the current PA scholarship, where researchers have predominantly worked with children in these areas. However, Skropanic’s study on graphic novels [86], which consists of similar production methods as animation, found this method palpable among older adult veterans to cope with posttraumatic stress disorder symptoms. While research continues to uncover animation’s efficacious role in promoting prohealth outcomes, regional perspectives may potentially stifle the implementation of PA among older populations. However, future participatory methods could include co-education or media literacy efforts as part of the rollout phase to normalize animation for adult audiences.

Animation remains widely used in professional settings, often requiring rigorously trained experts to create quality content. As such, it can be time-consuming, taking up to 10 weeks to craft a 60 to 90-second animation. However, the engagement between experts and community partners offers community-building opportunities, particularly with emerging technology. Nonprofit sectors have shown immense community-based engagement practices for animation. One such organization is Legends Animated, a nonprofit that explicitly hires animation apprentices of diverse backgrounds to provide valuable experiential learning and needed client-based artwork for their job portfolio [84]. They foster the capacity to create and work alongside the community sectors and stakeholders to develop animation relevant to their cultural experience. Software like Adobe Animate and Visem could unite diverse talent and voices of varying technological competencies, further democratizing the research setting. It is also worth noting that recent digital animation programs may have a manageable learning curve, making them accessible to creators of varying technical and artistic expertise.

Some ethical and accessibility concerns should also be addressed. To ensure equitable participation from co-creation to dissemination, rotating leadership alongside consent processes, especially as diverse skill sets are leveraged, should be considered [57,66]. This includes potential anonymity in voiceover recordings and visual masking or team names within credits. Specific to accessibility, user-friendly design should be at the forefront of the co-creation process. These include closed captions, audio narration, diverse language translations, screener-compatible descriptions, and diverse device compatibility (eg, phone vs tablet aspect ratio).

Although PA holds promise, empirical evaluations, particularly randomized trials or quantitative effectiveness studies, remain scarce. Future work should evaluate the efficacy of PA through mixed methods or controlled studies to establish robust, generalizable findings. Meanwhile, Wilson, Canty, and Miller’s [31] meta-analysis revealed that animation was efficacious in promoting prohealth behaviors and attitudes among children and adults, garnering insights into its methodological potential in health sectors. While PA presents risks in development and implementation, its benefits outweigh the associated costs. This medium, which integrates both dynamic and engaging features, has the potential to have a long-lasting impact and be disseminated across various digital structures, making it multimedia deployable.

## Conclusions

PA is a promising strategy for health promotion. This method leverages the animation’s nuanced development procedure to procure essential health determinants while enhancing messages. It also empowers communities to participate in their health landscapes. Although animation offers multisensory components functional for participatory praxis, it can be limited due to logistical (eg, cost and time consumption) and technological constraints (competencies). Overall, animation can be the tool to unlock and reveal limitless worlds and multiple truths, where one’s imagination is necessary to realize one’s civic potential and even acknowledge and address urgent health concerns.

## Supplementary material

10.2196/72737 Multimedia Appendix 1 Framework for analyzing participatory animation: process and product dimensions.

## Abbreviations

DHI: digital-based health intervention
PA: participatory animation
SEM: socioecological model
