# Supplementary material for: Participatory Animation for Health Promotion in Digital-Based Health Interventions: Viewpoint on Methodology and Application
Source: Online J Public Health Inform. 2025 Dec 5;17:e72737. doi: 10.2196/72737 (PMC12680130; doi:10.2196/72737)
Supplement: Multimedia Appendix 1 [file ojphi-v17-e72737-s001.docx]

Appendix

Table 1. Framework for Analyzing Participatory Animation: Process and Product Dimensions

| Component | Description | Potential Analytic Methods | Example Tools or Outputs |
| --- | --- | --- | --- |
| 1. Co-Creation Process | Include activities and stages involving community partners during animation development. (e.g., icebreakers, community discussion) | - Process tracing - Reflexive Journaling - Field notes analysis - Partnership quality rubrics | - Storyboarding sessions - Co-written scripts - Voiceover development logs - Concept art designs |
| 1. Content Formulation | Translation of ideas into animated content, including visual, narrative beats, and auditory elements | - Discourse analysis - Visual narrative analysis - Cultural representation audit | - Final character design sheets - Background artwork - Dialogue scripts - Musical selection |
| 1. Community Representation | Ways in which community voices, identities, and lived experiences are visualized and heard | - Semiotic analysis - Positionality mapping - Idenitut narrative coding | - Voiceover recordings - Character perspectives and experiencee s - Choice of symbols and metaphors |
| 1. Final Product Evaluation | Assessment of completed animation’s impact or message clarity for intended audiences | - Audience analysis - Pre/post attitude surveys - Focus groups with viewers | - Survey results - Message comprehension score - Viewer feedback |
| 1. Dissemination and Viewer Access | Distribution and accessibility strategies for community use and broader reach | - Accessibility audits - Media impact tracking - Digital equity analysis | - Platform analytics - Captioning checklists - Translation records |

Table 2: Theoretical Contributions of Participatory Animation for DHI

| Animation Production Affordances in Assessing Health Determinants | | Animation’s Affordances in Conveying Health Information | | |
| --- | --- | --- | --- | --- |
| Dialogical Framework | Dialogical  Narrative Analysis | The Cognitive Theory of Multimedia Learning | The Cognitive Load Theory | The Social Cognitive Theory |
| - Supports intersubjective exchanges, negotiations, and contestations - Resolves the 'paradox of communication' in research settings. - Mitigates challenges within dominant narrative frameworks | - Identifies tensions in communication modes - Unveils the potential of stories in conveying vital information - Engages core participants, indirect connections, and broader audiences - Utilizes a three-tiered approach: core group, indirect connections, and broader audiences. | - Engages auditory and visual processes for deeper learning - Enhances knowledge acquisition through combined verbal and visual materials - Knowledge acquisition is effective in encouraging pro-health behaviors. | - Manages working memory load for adequate comprehension - Enables better content processing - Incorporates complementary text, signaling cues, and content congruence. | - Enhances modeling structures for observation and experience - Enhances procedural components beneficial for conveying behavioral-related health messages |
